# Estimating life expectancy and years of life lost for autistic people in the UK: a matched cohort study

**DOI:** 10.1016/j.lanepe.2023.100776

**Published:** 2023-11-23

**Authors:** Elizabeth O'Nions, Dan Lewer, Irene Petersen, Jude Brown, Joshua E.J. Buckman, Rebecca Charlton, Claudia Cooper, CÉline El Baou, Francesca Happé, Jill Manthorpe, Douglas G.J. McKechnie, Marcus Richards, Rob Saunders, Cathy Zanker, Will Mandy, Joshua Stott

**Affiliations:** a UCL Research Department of Clinical, Educational and Health Psychology, Division of Psychology and Language Sciences, 1 – 19 Torrington Place, London, WC1E 7HB, UK; b Institute of Epidemiology and Healthcare, University College London, 1-19 Torrington Place, London, WC1E 7HB, UK; c Bradford Institute for Health Research, Bradford Teaching Hospitals NHS Foundation Trust, Duckworth Lane, Bradford, BD9 6RJ, UK; d UCL Research Department of Primary Care and Population Health, UCL Medical School (Royal Free Campus), Rowland Hill Street, London, NW3 2PF, UK; e National Autistic Society, 393 City Rd, London, EC1V 1NG, UK; f iCope – Camden & Islington NHS Foundation Trust, St Pancras Hospital, London, NW1 0PE, UK; g Department of Psychology, Goldsmiths University of London, New Cross, London, SE14 6NW, UK; h Queen Mary University of London, Centre for Psychiatry and Mental Health, Wolfson Institute of Population Health, London, E1 2AD, UK; i Social, Genetic and Developmental Psychiatry Centre, Institute of Psychiatry, Psychology and Neuroscience, King's College London, Memory Lane, London, SE5 8AF, UK; j NIHR Health & Social Care Workforce Research Unit, King's College London, Strand, London, WC2R 2LS, UK; k MRC Unit for Lifelong Health and Ageing at UCL, 1-19 Torrington Place, London, WC1E 7HB, UK; l London, UK

**Keywords:** Autism, Intellectual disability, Premature mortality, Life expectancy

## Abstract

**Background:**

Previous research has shown that people who have been diagnosed autistic are more likely to die prematurely than the general population. However, statistics on premature mortality in autistic people have often been misinterpreted. In this study we aimed to estimate the life expectancy and years of life lost experienced by autistic people living in the UK.

**Methods:**

We studied people in the IQVIA Medical Research Database with an autism diagnosis between January 1, 1989 and January 16, 2019. For each participant diagnosed autistic, we included ten comparison participants without an autism diagnosis, matched by age, sex, and primary care practice. We calculated age- and sex-standardised mortality ratios comparing people diagnosed autistic to the reference group. We used Poisson regression to estimate age-specific mortality rates, and life tables to estimate life expectancy at age 18 and years of life lost. We analysed the data separately by sex, and for people with and without a record of intellectual disability. We discuss the findings in the light of the prevalence of recorded diagnosis of autism in primary care compared to community estimates.

**Findings:**

From a cohort of nearly 10 million people, we identified 17,130 participants diagnosed autistic without an intellectual disability (matched with 171,300 comparison participants), and 6450 participants diagnosed autistic with an intellectual disability (matched with 64,500 comparison participants). The apparent estimates indicated that people diagnosed with autism but not intellectual disability had 1.71 (95% CI: 1.39–2.11) times the mortality rate of people without these diagnoses. People diagnosed with autism and intellectual disability had 2.83 (95% CI: 2.33–3.43) times the mortality rate of people without these diagnoses. Likewise, the apparent reduction in life expectancy for people diagnosed with autism but not intellectual disability was 6.14 years (95% CI: 2.84–9.07) for men and 6.45 years (95% CI: 1.37–11.58 years) for women. The apparent reduction in life expectancy for people diagnosed with autism and intellectual disability was 7.28 years (95% CI: 3.78–10.27) for men and 14.59 years (95% CI: 9.45–19.02 years) for women. However, these findings are likely to be subject to exposure misclassification biases: very few autistic adults and older-adults have been diagnosed, meaning that we could only study a fraction of the total autistic population. Those who have been diagnosed may well be those with greater support needs and more co-occurring health conditions than autistic people on average.

**Interpretation:**

The findings indicate that there is a group of autistic people who experience premature mortality, which is of significant concern. There is an urgent need for investigation into the reasons behind this. However, our estimates suggest that the widely reported statistic that autistic people live 16-years less on average is likely incorrect. Nine out of 10 autistic people may have been undiagnosed across the time-period studied. Hence, the results of our study do not generalise to all autistic people. Diagnosed autistic adults, and particularly older adults, are likely those with greater-than-average support needs. Therefore, we may have over-estimated the reduction in life expectancy experienced by autistic people on average. The larger reduction in life expectancy for women diagnosed with autism and intellectual disability vs. men may in part reflect disproportionate underdiagnosis of autism and/or intellectual disability in women.

**Funding:**

10.13039/501100000377Dunhill Medical Trust, 10.13039/501100000265Medical Research Council, 10.13039/501100000272National Institute for Health and Care Research, and the 10.13039/100010248Royal College of Psychiatrists.

**Research in context**

**Evidence before this study**

We searched PubMed from database inception to January 10th, 2023 using the search terms: (1) ‘autis∗’, and (2) ‘death’ or ‘mortality’ or ‘life-expectancy’ or ‘life expectancy’, without language restrictions. This identified articles presenting information about premature mortality in autistic people using electronic health records in high income countries. Studies varied considerably in terms of diagnosed autism prevalence within the source database, the proportion of autistic participants with co-occurring intellectual disability (ID), and the age of the participants included. The reliance on using electronic health records to identify diagnosed autistic people means that differential exposure misclassification is likely to have biased the results, particularly for studies of mortality in autistic adults. Few autistic adults have a diagnosis and those who do are likely have greater-than-average support needs. A meta-analysis of 12 studies of mortality in autistic people published in 2022 indicated higher rates of premature mortality in autistic females vs. males, and in autistic people with vs. without co-occurring ID. Meta-analytic results for studies including cause of death showed that diagnosed autistic people experienced increased mortality due to natural causes, accidents, and suicides. Only one study estimated the life-expectancy or life expectancy deficit experienced by autistic people, published in the US in 1998. Information on average-age-at-death for autistic vs. matched comparison people reported in at least two cohort studies has been misinterpreted as indicative of the life-expectancy deficit experienced by autistic people.

**Added value of this study**

This study aimed to estimate the difference in life expectancy for diagnosed autistic people in the UK compared to the general population. In the process of the analysis, we reached the conclusion that estimates from electronic health records may overestimate the reduction in life expectancy experienced by autistic people on average: only around 1 in 450 people had a diagnosis, despite the true number of autistic people being much higher. Males and females with a diagnosis of autism but not intellectual disability had an apparent 6-year shorter life-expectancy than people without a diagnosis. The apparent life expectancy for females with a diagnosis of autism and intellectual disability was nearly 15 years less than those with neither diagnosis, and for males with autism and intellectual disability, 7 years shorter. We speculate that the vast majority of autistic adults with few support needs were undiagnosed. Therefore, the true reduction in life expectancy may be less than the figures reported.

**Implications of all the available evidence**

Some autistic people experience a shorter life expectancy. This represents an inequity that could be reduced by alterations in health and social policy that improve support for and inclusion of autistic people. However, the reduction in life expectancy experienced by autistic people on average is likely to be less than the widely-reported figure of 16 years.

## Introduction

Autism is a lifelong neurodevelopmental condition that is present from birth, and affects how a person relates to others and perceives the world. Diagnostic criteria include the presence of difficulties with social communication and social interaction, and restricted and repetitive patterns of behaviours, interests, and activities.^1^ Between 1% and 3% of the population are autistic^o^.^2^^,^^3^ Autism is a spectrum condition: those diagnosed have widely varying support needs.

In many countries, younger people are more likely to have received an autism diagnosis compared to older people,^4^, ^5^, ^6^ reflecting greater recognition of autism and access to diagnostic services for children and young people in recent years. However, population surveys suggest that the actual prevalence of autism is similar across age-groups,^4^^,^^7^^,^^8^ implying that many adults, and particularly older adults, are undiagnosed. Access to adult diagnostic assessment services in the UK remains poor,^5^^,^^6^ with at least 9 out of 10 autistic over 50s in England estimated to be undiagnosed as of the end 2018.^6^

Adults who do have a diagnosis, particularly those who are middle-aged and older, are likely to have different support needs than autistic people who are undiagnosed. In some cases, the support needs of undiagnosed autistic adults may be considerable (e.g., the high proportion of undiagnosed autistic homeless people or undiagnosed autistic people with an eating disorder).^9^^,^^10^ However, on average we suspect that the support needs of diagnosed autistic people are probably greater than those who are undiagnosed (i.e. there is a differential exposure misclassification).^4^^,^^11^ For this reason, “one should be wary of passive studies that compare autistic people with their non-autistic peers, such as those using electronic health care records to compare rates of physical and mental health conditions”.^12^^,p.5^

Studies suggest that people diagnosed autistic are more likely than the general population to experience co-occurring neurodevelopmental challenges, including epilepsy, attention deficit hyperactivity disorder (ADHD), hearing or visual impairments, cerebral palsy, and intellectual disability (ID),^13^ though many autistic people do not have these conditions, and ascertainment bias may inflate the magnitude of differences. Poorer physical and mental health, including higher rates of psychiatric conditions, suicide attempts, immune conditions, gastrointestinal and sleep disorders, obesity, dyslipidemia, heart disease, and diabetes, have also been reported.^13^^,^^14^

Studies in both diagnosed and undiagnosed autistic people indicate that they are disproportionately likely to experience social adversity, discrimination, and a lack of support.^15^, ^16^, ^17^, ^18^, ^19^, ^20^ The effects of these experiences on physical and mental health, together with barriers to healthcare access^21^ and differences in health behaviour^22^ are likely to contribute to poorer health outcomes for autistic people on average, though the magnitude of these differences is unclear.

Whilst previous research has demonstrated that people diagnosed autistic are more likely to die prematurely,^23^ statistics on mortality risks have often been misinterpreted. A large Swedish study that followed diagnosed autistic people and an age-matched comparison sample over several years reported that the average age at death for the 2.6% of people diagnosed with autism but not ID who died during follow-up was 53.9 years, whilst for the 0.9% of comparison participants who died, the average value was 70.2 years.^24^ This 16.3-year difference has been widely described as the reduction in life expectancy experienced by autistic people.^25^, ^26^, ^27^ However, these figures reflect the average age at death for the small proportion of the sample in both the autistic and comparison groups who died. The young average age-at-death reflects the fact that most of those studied were young people, because young people are more likely to be diagnosed. Older autistic people are mostly undiagnosed, so are not represented in the sample.^6^ If a diagnosis is disproportionately made in younger people, the average age-at-death for deceased individuals during a short follow-up period will be young even when mortality rates are low.

A better method to estimate whether autistic people experience a reduced life expectancy compared to non-autistic people is the life table method, which is used by the UK Office for National Statistics (ONS) to estimate life expectancy.^28^ It involves calculating a mortality rate for each year of age, using the number of deaths in the age-group as the numerator and the person-time-at-risk in that age-group as the denominator. The age-specific rates are then used to estimate life expectancy. We found only one study that estimated the life expectancy deficit experienced by diagnosed autistic people using life tables. The study examined mortality in 11,347 autistic people living in California followed from 1980 to 1996.^29^ Compared to US national mortality statistics, life expectancy was reduced by 6.1 years for autistic men, and by 12.3 years for autistic women.^29^

Given the substantial widening of autism diagnostic criteria in the intervening time, we sought to update the life expectancy estimate based on more recent data from UK primary care electronic health records, and evaluate whether there is evidence to support the claim that autistic people live 16-years less on average. In this paper we provide the ‘naïve’ analyses of mortality and life expectancy and discuss if these estimates generalise to the whole autistic population.

## Method

### Study design

A matched retrospective cohort study.

### Setting

This study used UK electronic primary care health records from IQVIA Medical Research Data (IMRD). IQVIA Medical Research Data (IMRD) incorporates data from THIN, a Cegedim Database. Reference made to THIN is intended to be descriptive of the data asset licensed by IQVIA. IMRD contains anonymised electronic health records drawn from 794 UK primary care practices (c. 10% of all practices) including >18 million individuals, and is representative of the UK population.^30^

In the UK, almost all of the population are registered with an NHS primary care practice and access is free of charge.^31^ Non-emergency secondary and specialist care is mostly accessed via referral from a primary care practitioner (GP). Diagnoses made in secondary care (including autism), are communicated to the patient's GP.

### Ethical approval

IMRD holds ethical approval to collect and supply data for research purposes from the NHS London—South East Research Ethics Committee (reference 18/LO/0441). Use of the IMRD for this study was obtained and approved by IQVIA World Publications Scientific Review Committee in November 2022 (reference 22SRC034).

### Study population

We included two cohorts: people diagnosed with autism and ID, and people diagnosed with autism but not ID. Autism diagnoses were identified from the presence of a diagnostic label indicative of an autism spectrum condition (e.g., autism, Asperger's, pervasive developmental disorder) based on previously published studies.^32^^,^^33^ ID diagnoses were identified from the presence of a label indicative of intellectual disability (e.g., On learning disability register, Learning disability NOS), based on previously published studies.^6^^,^^32^^,^^33^ Code-lists are available as a Supplemental file. Cohort entry dates were defined based on the period of time when contributing general practices met quality criteria for both acceptable computer usage^34^ and acceptable mortality recording.^35^ The cohort entry date was the latest of the following dates: the date of diagnosis, the point at which quality criteria were met, registration at the practice +6 months, the practice contributing data to IMRD, and January 1, 1989; and cohort exit date was the earliest of death, deregistration from the practice, the practice no longer contributing data to IMRD, or January 16, 2019. This approach assumes that someone who is diagnosed autistic can be considered autistic for the rest of their life, but does not include time prior to diagnosis to avoid inclusion of immortal time in the analysis.

For each individual with an autism diagnosis, we sampled ten age-, sex-, and general-practice-matched comparison individuals. We used ‘exposure density sampling’ to identify the matched comparison groups and designate their cohort entry dates.^36^ For each individual joining the cohort who had been diagnosed autistic, we sampled comparison individuals from IMRD participants of the same age, sex, and registered at the same general practice, who did not have an autism or an ID diagnosis by that time; and then assigned them the same cohort entry date. eFigure S1 describes the identification of eligible records; and more information about exposure density sampling is provided in the Supplementary Methods and eFigure S2. Any participants diagnosed with autism or ID at any point prior to the date of registration +6 months would have been considered autistic/to have ID from the point of cohort entry.

### Study variables

The outcome was all-cause death. In the UK, registering of a death triggers an update in the NHS Personal Demographic Service, which is then automatically updated in the practice's electronic records. Prior to this system, GPs were notified about patient deaths by the local Health Authority, secondary care bereavement offices and/or by family members. Further information about identification of records indicating a death is provided in the Supplementary Methods.

Previous studies investigating mortality in autistic people have reported an increased likelihood of deaths from external causes, including suicide or accidents.^23^ We performed an additional search to identify possible deaths using a code-list to identify potentially fatal incidents, in case such deaths were not captured by the registration process described above. Further information is provided in the Supplementary Methods. We report results for analyses including these additional possible deaths as a sensitivity analysis.

To describe the sample, we also extracted data on: socioeconomic deprivation, genetic disorders; epilepsy/seizures; attention deficit and hyperactivity disorder; self-harm/suicide; severe mental illness; hearing and visual impairments; and severe mobility problems/cerebral palsy. For deceased individuals, we report the proportion who had ever had a code indicating stroke, cancer, dementia, heart disease (ischaemic heart disease and heart failure of any cause), or chronic obstructive pulmonary disease (COPD) for descriptive purposes. Code-lists are available as a Supplemental file.

Socioeconomic deprivation was measured using Townsend scores, derived for each area using 2001 census data.^37^ Each output area corresponds to approximately 150 households. For each area, statistics were calculated based on the percentage of households without access to a car, that were not owner-occupied, and household overcrowding, as well as the percentage of the economically active population aged 16–74 who were unemployed. For each variable, the scores were converted from exact scores into quintiles consisting of five groups of equal size to indicate local deprivation level. UK postcodes were then matched to output area Townsend deprivation quintile. A Townsend score of 1 indicates the least deprived quintile, and a score of 5, the most.

### Statistical analysis

We first calculated the number of deaths and person-years of observation, stratified by presence of an autism diagnosis, sex, and single-year-of-age from 18 to 100, accounting for time-varying age^p^.^38^ We then calculated the mortality rates in each stratum. We did this analysis separately for the cohorts of autistic people with and without an ID diagnosis and their respective comparison groups.

We used a Poisson model to estimate the mortality rate by single year-of-age in each group (autistic men, autistic women, comparison men, comparison women). The purpose of using a model rather than the observed rates is that we expected mortality rates to change smoothly with age, and this assumption adds power to the analysis. The dependent variable was the count of deaths and the independent variables were age (linear and quadratic terms) and an offset for the log observation time.

We used these modelled rates to estimate life expectancy at age 18 years using the period life table method described by the Office for National Statistics.^28^ We estimated 95% confidence intervals by simulating a distribution of mortality rates using the uncertainty in the Poisson model, and then estimating life expectancy for each simulation. Data preparation was performed using Stata 16, and data analyses using R version 4.2.2. Analysis code is available at https://github.com/danlewer/autism-life-expectancy. For ease of interpretation we report the total expected life expectancy at age 18 (i.e. we added 18 to the life expectancy at age 18).

To evaluate whether any observed differences in mortality might be attributable to the presence of co-occurring developmental/physical health conditions independently associated with high support needs and/or a reduced life expectancy, we adjusted the analysis estimating mortality ratios for these conditions based on diagnostic status at the start of follow-up.

### Patient and public involvement

Autistic people were involved in the design and conduct of this research. Four autistic adults who form part of an Experts by Experience Steering Group for this study and a representative from the UK National Autistic Society gave feedback on the appropriateness and usefulness of the research question addressed here. Their feedback informed the preparation of the manuscript. The National Autistic Society's Head of Evidence and Research (JB) was involved in the interpretation of the findings and the drafting of the manuscript.

### Role of the funding source

The funders of the study had no role in study design, data collection, data analysis, data interpretation, or writing of the paper.

## Results

### Participants

We identified 17,130 people with an autism diagnosis in their electronic health records without concurrent diagnosed ID and 6450 people diagnosed autistic with concurrent diagnosed ID. A total of 9,928,260 people had no autism record at any time prior to or during follow-up (eFigure 1). Across the time-period studied, around 1 in 450 people had an autism diagnosis (0.23%), approximately 1 in 10 of the likely true number based on current diagnostic criteria.

Overall, 50% of the people with autism but without ID had a diagnosis before 2006 and 50% had a diagnosis after 2006 (IQR: 2001–2012). For people diagnosed with autism and ID, 50% had a diagnosis before 2003 and 50% had a diagnosis after 2003 (IQR: 1997–2008). In general, those with a diagnosis of autism and ID were slightly older than those without ID, reflecting the fact that recording of ID tended to happen at an older age than the first record of diagnosed autism.

For participants with and without diagnosed ID respectively, the proportion that were male was 75.2% and 76.9% (Tables 1 and 2) and the proportions entering the study after 2010 were 66.8% and 76.8% respectively.

**Table 1:** Participant characteristics: people diagnosed with autism but not ID and their respective comparison groups.

	Men		Women	
	Diagnosed with autism but not ID	Comparison men	Diagnosed with autism but not ID	Comparison women
N individuals	13,172	131,720	3958	39,580
N practices	773	773	688	688
N deaths (%)	82 (0.62)	631 (0.48)	17 (0.43)	136 (0.34)
Median age at death for decedents (IQR)	48.97 (28.06–64.62)	49.53 (27.93–64.00)	51.89 (31.02–76.24)	55.50 (42.54–75.22)
**Age at cohort entry**				
Median age at entry (IQR)	18.96 (18.00–25.96)	18.96 (18.00–25.96)	20.63 (18.00–28.91)	20.63 (18.00–28.91)
18–24 years	9613 (72.98)	96,130 (72.98)	2645 (66.83)	26,450 (66.83)
25–34 years	1769 (13.43)	17,690 (13.43)	614 (15.51)	6140 (15.51)
35–44 years	912 (6.92)	9120 (6.92)	367 (9.27)	3670 (9.27)
45–54 years	571 (4.33)	5710 (4.33)	239 (6.04)	2390 (6.04)
55–64 years	236 (1.79)	2360 (1.79)	70 (1.77)	700 (1.77)
65+ years	71 (0.54)	710 (0.54)	23 (0.58)	230 (0.58)
Median length of follow-up (IQR)	2.25 (0.88–4.54)	2.62 (1.18–5.54)	1.82 (0.77–3.69)	2.21 (0.93–4.54)
**Socioeconomic status**				
n Townsend score 1 (%)	2057 (15.62)	23,991 (18.21)	591 (14.93)	6923 (17.49)
n Townsend score 2 (%)	1916 (14.55)	22,066 (16.75)	625 (15.79)	6383 (16.13)
n Townsend score 3 (%)	2346 (17.81)	23,937 (18.17)	680 (17.18)	7119 (17.99)
n Townsend score 4 (%)	2227 (16.91)	21,696 (16.47)	635 (16.04)	6507 (16.44)
n Townsend score 5 (%)	1634 (12.41)	14,959 (11.36)	466 (11.77)	4578 (11.57)
n Townsend score missing (%)	2992 (22.71)	25,071 (19.03)	961 (24.28)	8070 (20.39)
**Co-occurring conditions**				
n epilepsy at start (%)	672 (5.10)	1276 (0.97)	235 (5.94)	372 (0.94)
n genetic condition at start (%)	197 (1.50)	702 (0.53)	55 (1.39)	236 (0.60)
n SMI at start (%)	695 (5.28)	654 (0.50)	262 (6.62)	192 (0.49)
n ADHD at start (%)	2048 (15.55)	2516 (1.91)	373 (9.42)	170 (0.43)
n self-harm/suicide at start (%)	908 (6.89)	2536 (1.93)	690 (17.43)	1852 (4.68)
n severe hearing impairments at start (%)	267 (2.03)	2277 (1.73)	110 (2.78)	593 (1.50)
n severe visual impairments at start (%)	86 (0.65)	342 (0.26)	34 (0.86)	123 (0.31)
n severe mobility problems at start (%)	119 (0.90)	323 (0.25)	40 (1.01)	95 (0.24)
**Year of cohort entry**				
1989–1999	144 (1.10)	1440 (1.10)	54 (1.36)	540 (1.36)
2000–2009	3051 (23.16)	30,510 (23.16)	720 (18.19)	7200 (18.19)
2010–2019	9977 (75.74)	99,770 (75.74)	3184 (80.44)	31,840 (80.44)

Note: Abbreviations: SMI = Severe mental illness.

**Table 2:** Participant characteristics: autistic people with ID and their respective comparison groups.

	Men		Women	
	Diagnosed with autism and ID	Comparison men	Diagnosed with autism and ID	Comparison women
N individuals	4850	48,500	1600	16,000
N practices	714	714	548	548
N deaths (%)	92 (1.90)	415 (0.86)	36 (2.25)	96 (0.60)
Median age at death for decedents (IQR)	49.51 (32.61–63.54)	58.50 (41.53–68.68)	54.54 (40.39–64.74)	56.96 (43.05–70.08)
**Age at cohort entry**				
Median age at entry (IQR)	22.50 (18.57–33.07)	22.50 (18.57–33.07)	24.48 (19.43–36.03)	24.48 (19.43–36.03)
18–24 years	2911 (60.02)	29,110 (60.02)	827 (51.69)	8270 (51.69)
25–34 years	831 (17.13)	8310 (17.13)	350 (21.88)	3500 (21.88)
35–44 years	541 (11.15)	5410 (11.15)	205 (12.81)	2050 (12.81)
45–54 years	360 (7.42)	3600 (7.42)	143 (8.94)	1430 (8.94)
55–64 years	152 (3.13)	1520 (3.13)	50 (3.13)	500 (3.13)
65+ years	55 (1.13)	550 (1.13)	25 (1.56)	250 (1.56)
Median length of follow-up (IQR)	3.10 (1.30–6.18)	3.45 (1.43–6.37)	2.94 (1.17–6.37)	3.05 (1.19–6.34)
**Socioeconomic status**				
n Townsend score 1 (%)	738 (15.22)	9014 (18.59)	242 (15.13)	3082 (19.26)
n Townsend score 2 (%)	806 (16.62)	8598 (17.73)	269 (16.81)	2705 (16.91)
n Townsend score 3 (%)	982 (20.25)	9017 (18.59)	331 (20.69)	2887 (18.04)
n Townsend score 4 (%)	776 (16.00)	8154 (16.81)	237 (14.81)	2706 (16.91)
n Townsend score 5 (%)	564 (11.63)	5555 (11.45)	198 (12.38)	2064 (12.90)
n Townsend score missing (%)	984 (20.29)	8162 (16.83)	323 (20.19)	2556 (15.98)
**Co-occurring conditions**				
n epilepsy at start (%)	985 (20.31)	510 (1.05)	398 (24.88)	171 (1.07)
n genetic condition at start (%)	377 (7.77)	250 (0.52)	165 (10.31)	98 (0.61)
n SMI at start (%)	519 (10.70)	336 (0.69)	186 (11.63)	100 (0.63)
n ADHD at start (%)	612 (12.62)	721 (1.49)	126 (7.88)	42 (0.26)
n self-harm/suicide at start (%)	265 (5.46)	1153 (2.38)	160 (10.00)	832 (5.20)
n severe hearing impairments at start (%)	153 (3.15)	860 (1.77)	68 (4.25)	263 (1.64)
n severe visual impairments at start (%)	78 (1.61)	135 (0.28)	49 (3.06)	50 (0.31)
n severe mobility problems at start (%)	194 (4.00)	136 (0.28)	82 (5.13)	42 (0.26)
**Year of cohort entry**				
1989–1999	57 (1.18)	570 (1.18)	27 (1.69)	270 (1.69)
2000–2009	1531 (31.57)	15,310 (31.57)	524 (32.75)	5240 (32.75)
2010–2019	3262 (67.26)	32,620 (67.26)	1049 (65.56)	10,490 (65.56)

Note: Abbreviations: SMI = Severe mental illness.

All co-occurring conditions we examined were more common among participants diagnosed autistic than in the comparison groups (Tables 1 and 2). When comparing participants diagnosed with autism with and without ID, epilepsy, genetic disorders, severe mobility problems/cerebral palsy, blindness/low vision, deafness/significant hearing impairment, and severe mental illnesses were all more common among those with diagnosed ID, and were also more common among women diagnosed with autism and ID than in men. Conversely, self-harm/suicidality and attention deficit hyperactivity disorder were more common among people diagnosed autistic but not with ID. Further demographic information stratified by sex is provided in Tables 1 and 2

### Mortality rates and ratios

**Fig. 1:**
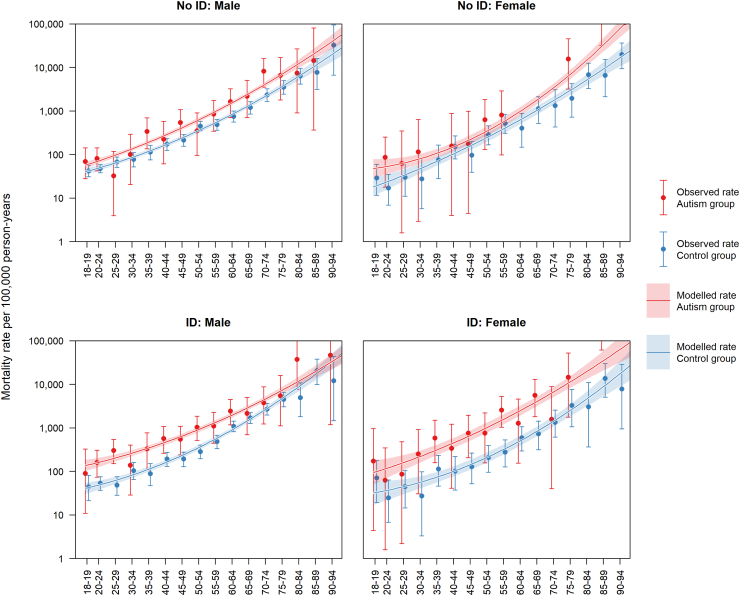
Mortality rate per 100,000 person-years for people diagnosed autistic vs. comparison participants. Error bars and shaded areas indicate 95% confidence intervals.

Among participants diagnosed with autism but not ID, 99/17,130 (0.58%) died, compared to 767/171,300 (0.45%) in the comparison group (see Table 1). Among participants diagnosed with autism and ID, 128/6450 (1.98%) died, compared to 511/64,500 (0.79%) in the comparison group (see Table 2). In all groups, we observed an approximate exponential increase in mortality rates with age (Fig. 1). People diagnosed with autism but not ID had 1.71 (95% CI: 1.39–2.11) times the mortality rate of people without these diagnoses. People diagnosed with autism and ID had 2.83 (95% CI: 2.33–3.43) times the mortality rate of people without these diagnoses. Stratifying the sample by sex, we found that women diagnosed with autism but not ID had 1.87 (95% CI: 1.12–3.10) times the mortality rate of comparison women, and men diagnosed with autism but not ID had 1.68 (95% CI: 1.34–2.12) times the mortality rate of comparison men. Women diagnosed with both autism and ID had 4.46 (95% CI: 3.03–6.57) times the mortality rate of comparison women, and men diagnosed with both autism and ID had 2.46 (95% CI: 1.96–3.08) times the mortality rate of comparison men. Mortality rates stratified by sex and age are provided in Table 3.

**Table 3:** Mortality rates per 100,000 person-years by age group and sex (with 95% CIs).

Age-band	Mortality rate per 100,000 person-years (Men)		Mortality rate per 100,000 person-years (Women)	
	Diagnosed with autism but not ID	Comparison men	Diagnosed with autism but not ID	Comparison women
18–24	76.17 (45.86–118.95)	45.68 (38.08–54.36)	51.20 (10.56–149.63)	21.44 (11.72–35.97)
25–34	54.61 (17.73–127.44)	70.42 (56.17–87.18)	80.97 (9.81–292.47)	29.17 (13.34–55.37)
35–44	285.17 (142.36–510.25)	141.42 (110.04–178.98)	81.24 (2.06–452.63)	114.26 (67.72–180.58)
45–54	460.52 (237.96–804.43)	315.84 (259.09–381.33)	383.98 (104.62–983.14)	182.35 (116.83–271.32)
55–64	1146.92 (641.92–1891.67)	586.41 (477.13–713.23)	538.18 (65.18–1944.07)	486.26 (308.25–729.63)
65+	4744.22 (2897.89–7327.07)	2298.76 (1937.45–2707.91)	4340.98 (1409.50–10130.39)	2714.06 (2001.13–3598.45)

Age-band	Diagnosed with autism and ID	Comparison men	Diagnosed with autism and ID	Comparison women
18–24	140.86 (70.32–252.04)	50.98 (36.74–68.91)	92.05 (11.15–332.52)	36.67 (15.83–72.26)
25–34	241.47 (132.01–405.14)	67.77 (48.19–92.64)	153.58 (31.67–448.82)	37.81 (15.20–77.90)
35–44	451.38 (246.77–757.34)	142.48 (103.11–191.92)	470.10 (172.52–1023.20)	107.62 (57.30–184.03)
45–54	756.34 (455.37–1181.12)	232.83 (178.10–299.08)	760.39 (305.72–1566.70)	164.24 (93.88–266.71)
55–64	1628.82 (948.85–2607.89)	730.81 (585.35–901.45)	2096.11 (958.48–3979.07)	393.62 (240.43–607.91)
65+	3912.70 (2279.29–6264.61)	2594.18 (2186.43–3055.91)	5338.00 (2440.88–10133.20)	1558.22 (1065.82–2199.74)

### Life expectancy

Our apparent estimated life expectancy for individuals diagnosed with autism but not ID was 76.84 years for women (95% CI: 72.23–81.49 years) and 74.57 years for men (95% CI: 71.94–77.60 years). This compared to 83.29 years (95% CI: 81.32–85.35) for matched comparison women and 80.71 years (95% CI: 79.46–82.11 years) for matched comparison men. Thus, women with diagnosed with autism but with no records of ID experienced 6.45 years-of-life lost (95% CI: 1.37–11.58 years), and men, 6.14 years (95% CI: 2.84–9.07 years).

**Fig. 2:**
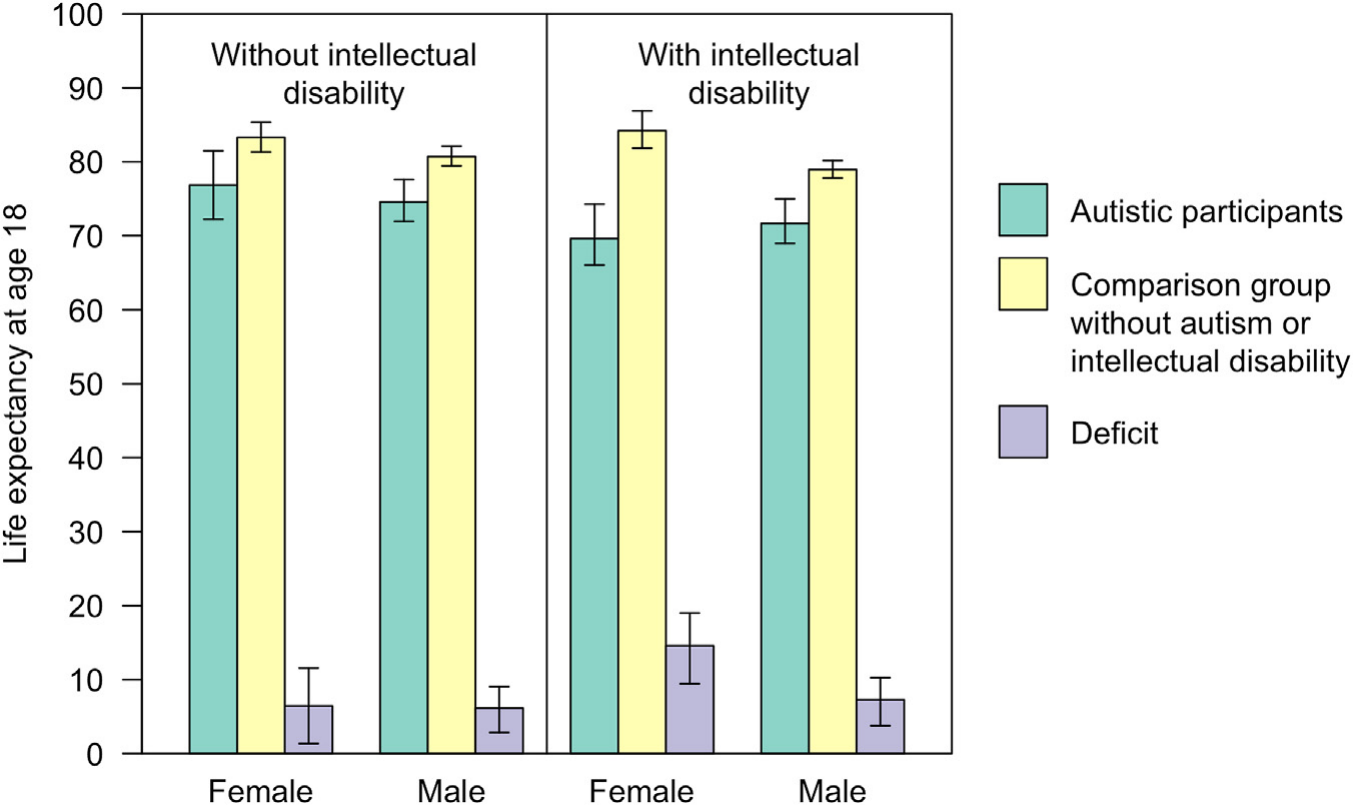
Life expectancy for participants diagnosed autistic with and without an intellectual disability, compared to matched comparison groups without a diagnosis of autism or ID. Error bars indicate 95% confidence intervals.

Life expectancy for participants diagnosed with autism and ID was 69.61 years for women (95% CI: 66.04–74.27 years), and 71.66 years for men (95% CI: 68.97–75.00 years). This compared to 84.20 years for comparison women (95% CI: 81.85–86.88 years) and 78.94 years (95% CI: 77.82–80.16 years) for comparison men. Thus, our apparent estimates suggest that having a diagnosis of autism and ID was associated with 14.59 years-of-life lost (95% CI: 9.45–19.02 years) in women and 7.28 years (95% CI: 3.78–10.27 years) in men. Life expectancies are presented in Fig. 2.

### Sensitivity analysis including definite and possible deaths

Including both definite and possible deaths had negligible impact on both the mortality ratios and the life expectancies and years-of-life lost experienced by people diagnosed autistic (see Supplementary eTable S6).

### Sensitivity analysis to estimate mortality ratios independent of co-occurring developmental/physical health conditions (definite deaths)

To estimate mortality in people diagnosed autistic independent of co-occurring conditions associated with high support needs and/or reduced life expectancy, we estimated Poisson models including the following variables as covariates: epilepsy; severe mobility problems; severe visual impairments; severe hearing impairments; genetic disorders; and ADHD (see Supplementary eTable S7).

After accounting for excess mortality associated with co-occurring conditions, women diagnosed with autism but not ID had 1.78 (95% CI: 1.07–2.98) times the mortality rate vs. comparison women, and men diagnosed with autism but not ID had 1.34 (95% CI: 1.05–1.71) times the mortality rate vs. comparison men. After accounting for excess mortality associated with co-occurring conditions, women diagnosed with autism and ID had 3.65 (95% CI: 2.32–5.72) times the mortality rate vs. comparison women, and men diagnosed with autism and ID had 1.91 (95% CI: 1.47–2.49) times the mortality rate vs. comparison men.

## Discussion

This analysis was motivated by the need to evaluate the claim that autistic people live 16 years less on average. This statistic, often cited in the mainstream press (e.g.,^39^) is hugely concerning for autistic people and their supporters. Our apparent estimate of years-of-life lost for women and men diagnosed with autism but not ID was around 6 years. For women with a record of both diagnosed autism and ID, the difference was approximately 15 years, and for men it was 7 years. The wide confidence intervals reflect the uncertainty around these estimates, due to the relatively small proportion of people with an autism diagnosis in the dataset, and the fact that most are young, meaning that there were only a small number of deaths.

Given that the identified reduction in life expectancy was lower than 16 years for autistic people without a learning disability, and epidemiological research indicates that only around 1 in 10 autistic people have at least a moderate learning disability,^7^ we believe that our findings suggest that the life expectancy deficit for autistic people on average is likely to be substantially lower than 16 years.

Although our apparent estimates indicate that in the UK, people who have a record of diagnosed autism in their primary care electronic health records experience a reduced life expectancy, we are aware that underdiagnosis means that our data is missing a significant proportion of autistic people. Over the time-period studied, we estimate that only around 1 in 10 people who would meet current diagnostic criteria for autism had been diagnosed autistic.

Most adults grew up at a time when autism was not widely applied as a diagnosis, as diagnostic criteria were highly restrictive and awareness of autism was limited.^40^ A 2007 study involving >7000 adults without intellectual disability surveyed participants to see if they met criteria for autism. Of the 19 who met criteria, none had a formal autism diagnosis.^4^ Similar surveys of patients from mental health outpatient and inpatient services reported that only a small fraction of research-identified autistic people had a pre-existing diagnosis.^41^^,^^42^ Therefore, in the present study, we believe that autism diagnostic records were not simply missing from the database, but were absent because diagnoses had not been made.

Across the time period studied, undiagnosed autistic adults may have been disproportionately those with fewer support needs who live and work in the community. An analysis of studies on diagnosed autistic adults published from 2000 to 2011 found that the average percentage of autistic adults who had a “good outcome”, or were living independently or semi-independently, was 20% on average.^43^ This contrasts with contemporaneous population-based work, which indicated that 9 out of 10 autistic adults had no or mild ID, were living independently or semi-independently in the community,^7^ and were not significantly less likely to be employed than the general population, though they did show lower rates of educational attainment.^4^ The apparent discrepancy between diagnosed and undiagnosed groups suggests that at least some of the observed excess mortality may be due to differential exposure misclassification: people with higher support needs and/or co-occurring neurodevelopmental, mental, or physical health conditions, who have more contact with services, were more likely to have been identified and diagnosed autistic. However, as noted in the introduction, some undiagnosed autistic people may have co-occurring conditions (e.g., eating disorders) or circumstances (e.g., homelessness) that put them at risk of premature mortality and overshadow their autism, meaning that it is not recognised and diagnosed.

### Comparison with other studies

Our estimates are similar to a previous study (now 25 years old) that estimated life expectancy in people diagnosed autistic,^29^ which reported a life expectancy deficit of 6.1 years for men and 12.3 years for women (this study did not include uncertainty ranges for these estimates). Notably, diagnostic criteria were considerably narrower at the time, reflected in much lower prevalence estimates; and exposure misclassification (i.e. underdiagnosis of autistic women) may also have contributed to the sex difference in estimated years-of-life-lost.

The mortality ratios in the present study resemble those from a recent meta-analysis, suggesting that premature mortality experienced by people diagnosed autistic in the UK may be similar to that in other high-income countries.^23^ However, other studies may be similarly affected by exposure misclassification, meaning that estimates may not generalise to all autistic people.

Our findings suggest that premature mortality was most evident in women with diagnosed autism and ID. Increased mortality has also been reported in women with ID compared to men irrespective of co-occurring autism.^44^ One explanation is that autism and ID are disproportionately underdiagnosed in women,^45^ meaning that only those with the very highest support needs or more co-occurring behavioural challenges^46^ get a diagnosis. Epidemiological surveys indicate that the male to female ratio for autistic people with moderate to severe/profound ID is around 1.2:1.^4^^,^^7^ In our data, we found a male to female ratio of 3:1 for autistic people with ID, suggesting disproportionate underdiagnosis in autistic women with ID.

Certain genetic disorders, such as Down syndrome,^44^ plus neurological conditions such as epilepsy,^44^^,^^47^ and profound and multiple disabilities^48^ are associated with a reduced life expectancy. If more women diagnosed with autism and ID had these conditions vs. men, this could explain the sex difference in the life expectancy deficit. In the present study, 10.3% of women with diagnosed autism and ID had a genetic/congenital condition and 24.9% had epilepsy or seizures at the start of follow-up, compared to 7.8% and 20.3% of men. Adjusting for epilepsy, genetic disorders, and other conditions associated with greater support needs only slightly reduced the sex difference in the mortality ratio, similar to previous findings in people with ID, suggesting that this does not fully account for the difference.^44^

Another possible explanation for the sex difference is that women diagnosed with autism and ID, as well as being disproportionately those with more support needs, experience more adversity leading to premature mortality; or a poorer standard of care compared to men (e.g., delayed diagnosis, misdiagnosis).^40^^,^^45^^,^^49^ Rates of self-harm/suicidality were nearly twice as high in women diagnosed with autism and ID vs. men (10.0% vs. 5.5%), indicating disproportionately unmet mental health needs.

Another potential contributing factor is that comparison male participants from the general population may experience a higher mortality rate than comparison female participants due to higher-risk lifestyles (e.g., smoking, drinking, road-traffic accidents), which may not be the case for people with ID who often lack the funds or opportunity to engage in these activities.^47^ Overall, our findings highlight the pressing need to disentangle the range of factors that potentially contribute to the sex-difference in years-of-life lost for women vs. men in those diagnosed with autism and ID.

Our mortality estimates for men and women diagnosed with autism and ID overlap with those reported for people with ID irrespective of autism in England from 2009 to 2013 (hazard ratio: 4.1 for women (95% CI: 3.6–4.7), and 3.3 for men (95% CI: 3.0–3.7)).^44^ However, our results differ from those reported by the English LeDeR study. Comparing average age at death for the 3304 people with ID who were notified to the LeDeR programme to national mortality data, the study reported that men with ID died 22 years younger, and women with ID 26 years younger, than people without ID from the general population during 2021.^50^ The large discrepancy compared to our estimates of years-of-life-lost may partly be because average age at death encompasses mortality in people with ID and the age distribution of people in the population who have an ID diagnosis, which is affected by changes in diagnostic practices. Another factor could be differences in participant characteristics: deceased individuals in the LeDeR study might overrepresent people with more co-occurring conditions who died younger than deceased individuals in the present study.

### Relevance for policy and research

Our findings indicate that, in the UK, there is a group of diagnosed autistic people who experience a reduced life expectancy, which is extremely concerning. This is in line with recent reports about failures of care and inadequate support leading to avoidable deaths in autistic people and people with ID.^39^^,^^50^^,^^51^

Existing evidence indicates that autistic people and people with intellectual disabilities disproportionately experience forms of adversity linked to poorer physical and mental wellbeing, such as victimisation,^15^^,^^20^ discrimination,^16^^,^^52^^,^^53^ inadequate support,^39^^,^^54^ unmet mental health needs,^54^^,^^55^ inappropriate or inadequate care,^39^^,^^51^^,^^56^^,^^57^ homelessness,^58^ and poor access to healthcare.^21^^,^^59^ Given their association with poorer health and outcomes, these factors may contribute significantly to the lower life expectancy, yet they are not captured in primary care records. For example, difficulties accessing care or communicating with healthcare providers^21^ can lead to delayed identification of common conditions such as constipation or depression, increasing the risk of mortality.^57^^,^^60^

Many long-term health conditions are impacted by adverse life experiences. For example, lack of opportunities to participate in community activities, disproportionately experienced by some autistic people^19^^,^^61^ are linked to poverty and loneliness,^62^ which are linked to poor mental health.^63^^,^^64^ Unemployment and lack of access to community activities are also linked to sedentary behaviour,^65^, ^66^, ^67^ which is linked to obesity and cardiovascular disease, also more common in people diagnosed autistic.^14^ These issues could be addressed with better service design to address systemic biases that discriminate against autistic people, more social support, and enforcement of equality legislation.^68^

### Strengths and limitations

This study has a number of strengths and limitations. Both the diagnosed autistic and matched comparison participants were drawn from the same general practices, meaning that regional variation in mortality, or practice-level variability in mortality recording would have affected both equally. We were unable to link our data to ONS data, which provides the most complete record of deaths in the UK population available. However, a previous study using UK primary care data that was linked to ONS reported that 98.2% of deaths in ONS were also identified in primary care records.^69^ The life expectancies for the comparison groups closely resemble those from ONS life tables for the general population of the UK (c.80 years for men and c.83 years for women).^28^ Therefore we are confident that we have identified deaths with sufficient accuracy.

Limitations include the absence of information about cause of death, which meant that we could not explore how years of life lost for people diagnosed autistic is attributable to different causes. Population-based studies that identify diagnosed and undiagnosed autistic people in the community are needed to provide more conclusive evidence on life expectancy irrespective of diagnostic status. This is important because undiagnosed autistic people may experience specific risk factors for premature mortality, such as lack of support, problems at work, and financial exploitation.^15^ We were unable to explore differences in mortality experienced by gender diverse autistic people, as gender diversity was not coded in the database.

A further limitation is the lack of generalisability to other countries or time periods. This is partly due to changes in diagnostic criteria for autism, and partly due to changes in factors that impact the health of autistic people, such as the accessibility of statutory support and primary care. It should also be noted that period life expectancy, which we report here, does not reflect the age that someone diagnosed with autism who is 18 years old today would expect to die—instead it is a summary of contemporary mortality rates. People who are now aged 18 would be expected to live longer due to medical advances in the future.

### Conclusions and implications

This study aimed to estimate the difference in life expectancy for diagnosed autistic people compared to the general population. Our findings suggest that the reduction in life expectancy experienced by autistic people on average is likely to be substantially less than the widely-reported figure of 16 years. However, the results show that some autistic people do die prematurely, representing an inequity that could be reduced by better support, inclusion, and changes that facilitate access to services. e.g.,^70^

People diagnosed autistic but not with intellectual disability had an apparent 6-year shorter life-expectancy. The apparent reduction in life expectancy for women diagnosed autistic with an intellectual disability was nearly 15 years, and for men, 7 years. We speculate that these estimates may overestimate the reduction in life expectancy experienced by autistic people on average. This is because only around 1 in 450 people had an autism diagnosis, despite the true number of autistic people being much higher. Those with few support needs were likely disproportionately undiagnosed. Therefore, the true reduction in life expectancy experienced by autistic people on average may be lower than the figures presented.

## Contributors

EO and DL conceived of the study. EO and IP accessed and verified the data. EO and DL undertook the analysis. All authors interpreted the findings. EO and DL wrote the first draft of the manuscript; all other authors revised the manuscript for critically important content and approved the final version. The corresponding author attests that all listed authors meet authorship criteria and that no others meeting the criteria have been omitted. All authors accept responsibility to submit for publication.

## Data sharing statement

Individual participant data cannot be shared.

## Ethical approval

IMRD holds ethical approval to collect and supply data for research purposes from the NHS London—South East Research Ethics Committee (reference 18/LO/0441). Use of the IMRD for this study was obtained and approved by IQVIA World Publications Scientific Review Committee on 21st September 2022 (reference 22SRC034).

## Declaration of interests

DL, IP, RC, CC, CEB, FH, JM, JBr, RS, CZ, JS, & WM declare no support from any organisation for the submitted work. EO received a post-doctoral fellowship from the Dunhill Medical Trust which funded completion of the work. DM was supported by NIHR as an In-Practice Fellow [NIHR301988]. MR was supported by the Medical Research Council [MC_UU_00019/1] and [MC_UU_00019/3] and JBu was supported by the Royal College of Psychiatrists. JS received funding from the ESRC and NIHR. WM is involved in unrelated projects funded by ESRC, NIHR, MRC, ERC, Sarepta Therapeutics, and Autistica, and received royalties from Jessica Kingsley publishers and a staff training fee from Jazz Pharma. FH is part-funded by the NIHR Biomedical Research Centre at South London and Maudsley NHS Foundation Trust and King's College London. RC received funding from NIHR. DM has received payment from EMIS/patient. info for writing patient- and professional-facing material for topics unrelated to this manuscript.

All authors declare that they have no financial relationships with any organisations that might have an interest in the submitted work in the previous three years, and no other relationships or activities that could appear to have influenced the submitted work. The views expressed are those of the authors and not necessarily those of the NHS, the NIHR or the Department of Health and Social Care.

## Appendix A Supplementary data

Supplementary eTables S1–S7

Codelists

Supplementary eFigures S1–S3

## Footnotes

o We use identity-first langage (i.e. “autistic person”) based on guidelines published by the National Autistic Society.

p This means that an individual who joins on their 18th birthday and is followed for 3 years contributes 1 year at age 18, 1 year at age 19, and 1 year at age 20.
