# Supplementary material for: Estimating life expectancy and years of life lost for autistic people in the UK: a matched cohort study
Source: Lancet Reg Health Eur. 2023 Nov 23;36:100776. doi: 10.1016/j.lanepe.2023.100776 (PMC10769892; doi:10.1016/j.lanepe.2023.100776)
Supplement: Supplementary eTables S1–S7 [file mmc1.docx]

**Supplementary Tables for “Estimating life expectancy and years of life lost for autistic people in the UK: a matched cohort study”**

Contents

[eTable 1: Demographics of deceased autistic people without ID and deceased people from the comparison group (main analysis; definite deaths) 2](#_Toc150336088)

[eTable 2: Demographics of deceased autistic people with ID and deceased people from the comparison group (main analysis; definite deaths) 3](#_Toc150336089)

[eTable 3: Demographics of deceased autistic people without ID and deceased people from the comparison group (sensitivity analysis; definite and possible deaths) 4](#_Toc150336090)

[eTable 4: Demographics of deceased autistic people with ID and deceased people from the comparison group (sensitivity analysis; definite and possible deaths) 5](#_Toc150336091)

[eTable 5: Crude mortality rates by sex and age-band: possible and definite deaths 6](#_Toc150336092)

[eTable 6: Estimated life expectancy including (A) only definite deaths, and (B) possible and definite deaths 7](#_Toc150336093)

[eTable 7: Mortality ratios adjusted for co-occurring developmental conditions: definite deaths 8](#_Toc150336094)

## eTable 1: Demographics of deceased autistic people without ID and deceased people from the comparison group (main analysis; definite deaths)

|  | Deceased autistic people without ID | Deceased people from the comparison group |
| --- | --- | --- |
| N individuals (% of cohort) | 99 (0.58) | 767 (0.45) |
| N practices | 93 | 360 |
| Median age at entry (IQR) | 46.80 (25.76 - 59.46) | 45.60 (23.83 - 58.79) |
| Median age at death (IQR) | 49.59 (28.06 - 65.53) | 51.13 (29.25 - 64.98) |
| n males (%) | 82 (82.83) | 631 (82.27) |
| n females (%) | 17 (17.17) | 136 (17.73) |
| *Socioeconomic deprivation* | | |
| n Townsend score 1 (%) | 12 (12.12) | 115 (14.99) |
| n Townsend score 2 (%) | 17 (17.17) | 142 (18.51) |
| n Townsend score 3 (%) | 22 (22.22) | 144 (18.77) |
| n Townsend score 4 (%) | 13 (13.13) | 133 (17.34) |
| n Townsend score 5 (%) | 11 (11.11) | 111 (14.47) |
| n Townsend score missing (%) | 24 (24.24) | 122 (15.91) |
| *Co-occurring conditions* | | |
| n epilepsy ever (%) | 14 (14.14) | 43 (5.61) |
| n genetic condition ever (%) | ** (<6) | 11 (1.43) |
| n SMI ever (%) | 25 (25.25) | 35 (4.56) |
| n ADHD ever (%) | ** (<6) | 12 (1.56) |
| n suicide/SH ever (%) | 23 (23.23) | 80 (10.43) |
| n severe hearing ever (%) | ** (<6) | 46 (6.00) |
| n severe visual ever (%) | ** (<6) | 17 (2.22) |
| n severe mobility problems ever (%) | ** (<6) | 32 (4.17) |
| n cancer ever (%) | 20 (20.20) | 210 (27.38) |
| n COPD ever (%) | 7 (7.07) | 64 (8.34) |
| n dementia ever (%) | ** (<6) | 17 (2.22) |
| n heart disease/ failure ever (%) | 12 (12.12) | 103 (13.43) |
| n stroke ever (%) | 9 (9.09) | 47 (6.13) |
| *Age at cohort entry* | | |
| 18 - 24 years | 24 (24.24) | 194 (25.29) |
| 25 - 34 years | 8 (8.08) | 78 (10.17) |
| 35 - 44 years | 12 (12.12) | 100 (13.04) |
| 45 - 54 years | 22 (22.22) | 151 (19.69) |
| 55 - 64 years | 14 (14.14) | 111 (14.47) |
| 65+ years | 19 (19.19) | 133 (17.34) |

Note: ** indicates information redacted. SMI: Severe mental illness; SH: self-harm; COPD: chronic obstructive pulmonary disease; “Ever” includes records after the date of death.

## eTable 2: Demographics of deceased autistic people with ID and deceased people from the comparison group (main analysis; definite deaths)

|  | Deceased autistic people with ID | Deceased people from the comparison group |
| --- | --- | --- |
| N individuals (% of cohort) | 128 (1.98) | 511 (0.79) |
| N practices | 105 | 239 |
| Median age at entry (IQR) | 45.17 (33.49 - 56.64) | 52.50 (35.94 - 63.89) |
| Median age at death (IQR) | 51.42 (35.71 - 63.92) | 58.45 (41.64 - 69.30) |
| n males (%) | 92 (71.88) | 415 (81.21) |
| n females (%) | 36 (28.13) | 96 (18.79) |
| *Socioeconomic deprivation* | | |
| n Townsend score 1 (%) | 12 (9.38) | 59 (11.55) |
| n Townsend score 2 (%) | 25 (19.53) | 83 (16.24) |
| n Townsend score 3 (%) | 24 (18.75) | 96 (18.79) |
| n Townsend score 4 (%) | 22 (17.19) | 92 (18.00) |
| n Townsend score 5 (%) | 23 (17.97) | 99 (19.37) |
| n Townsend score missing (%) | 22 (17.19) | 82 (16.05) |
| *Co-occurring conditions* | | |
| n epilepsy ever (%) | 40 (31.25) | 17 (3.33) |
| n genetic condition ever (%) | 16 (12.50) | 6 (1.17) |
| n SMI ever (%) | 25 (19.53) | 27 (5.28) |
| n ADHD ever (%) | ** (<5) | ** (<1.17) |
| n suicide/SH ever (%) | 15 (11.72) | 67 (13.11) |
| n severe hearing ever (%) | 6 (4.69) | 28 (5.48) |
| n severe visual ever (%) | 9 (7.03) | 7 (1.37) |
| n severe mobility problems ever (%) | 22 (17.19) | 21 (4.11) |
| n cancer ever (%) | 14 (10.94) | 154 (30.14) |
| n COPD ever (%) | ** (<5) | 65 (12.72) |
| n dementia ever (%) | 7 (5.47) | 10 (1.96) |
| n heart disease/ heart failure ever (%) | ** (<5) | 98 (19.18) |
| n stroke ever (%) | 7 (5.47) | 43 (8.41) |
| *Age at cohort entry* | | |
| 18 - 24 years | 19 (14.84) | 74 (14.48) |
| 25 - 34 years | 18 (14.06) | 49 (9.59) |
| 35 - 44 years | 26 (20.31) | 74 (14.48) |
| 45 - 54 years | 27 (21.09) | 85 (16.63) |
| 55 - 64 years | 19 (14.84) | 119 (23.29) |
| 65+ years | 19 (14.84) | 110 (21.53) |

Note: ** indicates information redacted. SMI: Severe mental illness; SH: self-harm; COPD: chronic obstructive pulmonary disease. “Ever” includes records after the date of death.

## eTable 3: Demographics of deceased autistic people without ID and deceased people from the comparison group (sensitivity analysis; definite and possible deaths)

|  | Deceased autistic people without ID: possible and definite deaths | Deceased people from the comparison group: possible and definite deaths |
| --- | --- | --- |
| N individuals (% of cohort) | 108 (0.63) | 792 (0.46) |
| N practices | 101 | 369 |
| Median age at entry (IQR) | 45.22 (23.90 - 57.81) | 44.77 (22.97 - 58.49) |
| Median age at death (possible & definite) (IQR) | 48.54 (25.74 - 63.41) | 50.14 (28.29 - 64.62) |
| n males (%) | 88 (81.48) | 653 (82.45) |
| n females (%) | 20 (18.52) | 139 (17.55) |
| *Socioeconomic deprivation* | | |
| n Townsend score 1 (%) | 12 (11.11) | 117 (14.77) |
| n Townsend score 2 (%) | 17 (15.74) | 146 (18.43) |
| n Townsend score 3 (%) | 25 (23.15) | 144 (18.18) |
| n Townsend score 4 (%) | 13 (12.04) | 140 (17.68) |
| n Townsend score 5 (%) | 15 (13.89) | 118 (14.90) |
| n Townsend score missing (%) | 26 (24.07) | 127 (16.04) |
| *Co-occurring conditions* | | |
| n epilepsy ever (%) | 15 (13.89) | 42 (5.30) |
| n genetic/congenital condition ever (%) | ** (<6) | 11 (1.39) |
| n SMI ever (%) | 26 (24.07) | 37 (4.67) |
| n ADHD ever (%) | ** (<6) | 14 (1.77) |
| n suicide/SH ever (%) | 32 (29.63) | 106 (13.38) |
| n severe hearing ever (%) | ** (<6) | 47 (5.93) |
| n severe visual ever (%) | ** (<6) | 17 (2.15) |
| n severe mobility problems ever (%) | ** (<6) | 32 (4.04) |
| n cancer ever (%) | 20 (18.52) | 210 (26.52) |
| n COPD ever (%) | 7 (6.48) | 64 (8.08) |
| n dementia ever (%) | ** (<6) | 17 (2.15) |
| n heart disease/failure ever (%) | 12 (11.11) | 103 (13.01) |
| n stroke ever (%) | 9 (8.33) | 47 (5.93) |
| *Age at cohort entry* | | |
| 18 - 24 years | 29 (26.85) | 211 (26.64) |
| 25 - 34 years | 10 (9.26) | 83 (10.48) |
| 35 - 44 years | 14 (12.96) | 103 (13.01) |
| 45 - 54 years | 22 (20.37) | 151 (19.07) |
| 55 - 64 years | 14 (12.96) | 111 (14.02) |
| 65+ years | 19 (17.59) | 133 (16.79) |

Note: ** indicates information redacted. SMI: Severe mental illness; SH: self-harm; COPD: chronic obstructive pulmonary disease. “Ever” includes records after the date of death.

## eTable 4: Demographics of deceased autistic people with ID and deceased people from the comparison group (sensitivity analysis; definite and possible deaths)

|  | Deceased autistic people with ID: possible and definite deaths | Deceased people from the comparison group: possible and definite deaths |
| --- | --- | --- |
| N individuals (% of cohort) | 132 (2.05) | 520 (0.81) |
| N practices | 108 | 244 |
| Median age at entry (IQR) | 44.71 (31.60 - 56.50) | 52.20 (34.73 - 63.55) |
| Median age at death (possible & definite) (IQR) | 50.09 (34.25 - 63.71) | 57.84 (41.04 - 69.17) |
| n males (%) | 95 (71.97) | 421 (80.96) |
| n females (%) | 37 (28.03) | 99 (19.04) |
| *Socioeconomic deprivation* | | |
| n Townsend score 1 (%) | 12 (9.09) | 59 (11.35) |
| n Townsend score 2 (%) | 25 (18.94) | 83 (15.96) |
| n Townsend score 3 (%) | 26 (19.70) | 96 (18.46) |
| n Townsend score 4 (%) | 22 (16.67) | 97 (18.65) |
| n Townsend score 5 (%) | 23 (17.42) | 101 (19.42) |
| n Townsend score missing (%) | 24 (18.18) | 84 (16.15) |
| *Co-occurring conditions* | | |
| n epilepsy ever (%) | 42 (31.82) | 17 (3.27) |
| n genetic/congenital condition ever (%) | 18 (13.64) | 6 (1.15) |
| n SMI ever (%) | 26 (19.70) | 29 (5.58) |
| n ADHD ever (%) | 6 (4.55) | ** (< 1.15) |
| n suicide/SH ever (%) | 19 (14.39) | 76 (14.62) |
| n severe hearing ever (%) | 6 (4.55) | 28 (5.38) |
| n severe visual ever (%) | 9 (6.82) | 7 (1.35) |
| n severe mobility problems ever (%) | 22 (16.67) | 21 (4.04) |
| n cancer ever (%) | 14 (10.61) | 154 (29.62) |
| n COPD ever (%) | ** (< 5) | 65 (12.50) |
| n dementia ever (%) | 7 (5.30) | 10 (1.92) |
| n heart disease/ failure ever (%) | ** (< 5) | 98 (18.85) |
| n stroke ever (%) | 7 (5.30) | 43 (8.27) |
| *Age at cohort entry* | | |
| 18 - 24 years | 22 (16.67) | 81 (15.58) |
| 25 - 34 years | 19 (14.39) | 50 (9.62) |
| 35 - 44 years | 26 (19.70) | 75 (14.42) |
| 45 - 54 years | 27 (20.45) | 85 (16.35) |
| 55 - 64 years | 19 (14.39) | 119 (22.88) |
| 65+ years | 19 (14.39) | 110 (21.15) |

Note: ** indicates information redacted. SMI: Severe mental illness; SH: self-harm; COPD: chronic obstructive pulmonary disease. “Ever” includes records after the date of death.

## eTable 5: Crude mortality rates by sex and age-band: possible and definite deaths

| Age-band | **Mortality rate per 100,000 (Females)** | | **Mortality rate per 100,000 (Males)** | |
| --- | --- | --- | --- | --- |
|  | Autistic females without ID | Comparison females | Autistic males without ID | Comparison males |
| 18 - 24 | 85.34 (27.71 to 199.15) | 22.97 (12.86 to 37.88) | 88.20 (55.28 to 133.54) | 51.08 (43.02 to 60.21) |
| 25 - 34 | 121.45 (25.05 to 354.93) | 35.65 (17.80 to 63.79) | 65.53 (24.05 to 142.63) | 72.93 (58.42 to 89.96) |
| 35 – 44 | 81.24 (2.06 to 452.63) | 114.26 (67.72 to 180.58) | 311.10 (160.75 to 543.43) | 149.61 (117.27 to 188.11) |
| 45 - 54 | 383.98 (104.62 to 983.14) | 182.35 (116.83 to 271.32) | 498.90 (265.64 to 853.14) | 315.84 (259.09 to 381.33) |
| 55 - 64 | 538.18 (65.18 to 1944.07) | 486.26 (308.25 to 729.63) | 1146.92 (641.92 to 1891.67) | 586.41 (477.13 to 713.23) |
| 65+ | 4340.98 (1409.50 to 10130.39) | 2714.06 (2001.13 to 3598.45) | 4744.22 (2897.89 to 7327.07) | 2298.76 (1937.45 to 2707.91) |
|  | Autistic females with ID | Comparison females | Autistic males with ID | Comparison males |
| 18 - 24 | 138.08 (28.48 to 403.53) | 41.26 (18.86 to 78.32) | 166.48 (88.64 to 284.68) | 55.84 (40.88 to 74.48) |
| 25 - 34 | 153.58 (31.67 to 448.82) | 48.61 (22.23 to 92.28) | 258.71 (144.80 to 426.71) | 67.77 (48.19 to 92.64) |
| 35 – 44 | 470.10 (172.52 to 1023.20) | 107.62 (57.30 to 184.03) | 451.38 (246.77 to 757.34) | 149.11 (108.76 to 199.52) |
| 45 - 54 | 760.39 (305.72 to 1566.70) | 164.24 (93.88 to 266.71) | 756.34 (455.37 to 1181.12) | 232.83 (178.10 to 299.08) |
| 55 - 64 | 2096.11 (958.48 to 3979.07) | 393.62 (240.43 to 607.91) | 1628.82 (948.85 to 2607.89) | 730.81 (585.35 to 901.45) |
| 65+ | 5338.00 (2440.88 to 10133.20) | 1558.22 (1065.82 to 2199.74) | 3912.70 (2279.29 to 6264.61) | 2594.18 (2186.43 to 3055.91) |

## eTable 6: Estimated life expectancy including (A) only definite deaths, and (B) possible and definite deaths

|  | **Female** | **Male** |
| --- | --- | --- |
| **Autism No ID** | | |
| **(A) Definite only** | | |
| Autism without ID | 76.84 (72.23, 81.49) | 74.57 (71.94, 77.60) |
| Comparison group | 83.29 (81.32, 85.35) | 80.71 (79.46, 82.11) |
| Difference | 6.45 (1.37, 11.58) | 6.14 (2.84, 9.07) |
| **(B) Definite + possible** | | |
| Autism without ID | 76.62 (71.93, 81.22) | 74.29 (71.72, 77.35) |
| Comparison group | 83.27 (81.37, 85.31) | 80.61 (79.41, 81.93) |
| Difference | 6.65 (1.69, 11.75) | 6.32 (3.07, 9.25) |
| **Autism With ID** | | |
| **(A) Definite only** | | |
| Autism with ID | 69.61 (66.04, 74.27) | 71.66 (68.97, 75.00) |
| Comparison group | 84.20 (81.85, 86.88) | 78.94 (77.82, 80.16) |
| Difference | 14.59 (9.45, 19.02) | 7.28 (3.78, 10.27) |
| **(B) Definite + possible** | | |
| Autism with ID | 69.38 (65.92, 73.82) | 71.47 (68.77, 74.71) |
| Comparison group | 84.12 (81.79, 86.73) | 78.88 (77.77, 80.07) |
| Difference | 14.74 (9.70, 19.05) | 7.41 (4.02, 10.42) |

## eTable 7: Mortality ratios adjusted for co-occurring developmental conditions: definite deaths

|  | **Adjusted SIRs (95% CI)** | |
| --- | --- | --- |
|  | **Females** | **Males** |
| **Autism without ID** | | |
| Autism | 1.78 (1.07 - 2.98) | 1.34 (1.05 - 1.71) |
| age (years) | 1.05 (1.01 - 1.09) | 1.04 (1.02 - 1.06) |
| age^2^ (years) | 1.00 (1.00 - 1.00) | 1.00 (1.00 - 1.00) |
| Epilepsy | 3.05 (1.46 - 6.38) | 3.13 (2.21 - 4.43) |
| Severe mobility problem | 4.14 (1.32 - 13.05) | 3.81 (2.21 - 6.55) |
| Severe visual impairment | 0.77 (0.23 - 2.55) | 3.73 (1.99 - 6.98) |
| Severe hearing impairment | 1.29 (0.68 - 2.46) | 1.11 (0.75 - 1.63) |
| Genetic disorder | 3.12 (0.98 - 9.89) | 2.21 (1.04 - 4.67) |
| ADHD | 0.00 (0.00 - 0.00) | 1.76 (1.03 - 2.99) |
| **Autism with ID** | | |
| Autism | 3.65 (2.32 - 5.72) | 1.91 (1.47 - 2.49) |
| age (years) | 1.01 (0.96 - 1.06) | 1.01 (0.99 - 1.04) |
| age^2^ (years) | 1.00 (1.00 - 1.00) | 1.00 (1.00 - 1.00) |
| Epilepsy | 0.98 (0.49 – 2.00) | 1.42 (0.96 - 2.09) |
| Severe mobility problem | 2.60 (1.23 - 5.52) | 3.52 (2.09 - 5.93) |
| Severe visual impairment | 3.50 (1.50 - 8.16) | 1.67 (0.74 - 3.76) |
| Severe hearing impairment | 0.74 (0.27 - 2.02) | 1.20 (0.78 - 1.86) |
| Genetic disorder | 1.93 (0.81 - 4.58) | 2.47 (1.42 - 4.30) |
| ADHD | 0.00 (0.00 - 0.00) | 1.06 (0.47 - 2.42) |
