# Supplementary material for: Estimating life expectancy and years of life lost for autistic people in the UK: a matched cohort study
Source: Lancet Reg Health Eur. 2023 Nov 23;36:100776. doi: 10.1016/j.lanepe.2023.100776 (PMC10769892; doi:10.1016/j.lanepe.2023.100776)
Supplement: Supplementary eFigures S1–S3 [file mmc3.docx]

**Supplementary Figures for “Estimating life expectancy and years of life lost for autistic people in the UK: a matched cohort study”**

Contents

[eFigure 1: Flow diagram for practice/ participant exclusions 2](#_Toc146039132)

[eFigure 2: Simplified schematic description of steps to apply EDS to identify the matched groups & start dates 3](#_Toc146039133)

[eFigure 3: Identification of deaths 5](#_Toc146039134)

## eFigure 1: Flow diagram for practice/ participant exclusions

| 794 practices |  | 2 excluded: Missing Acceptable Computer Usage/ Acceptable Mortality Recording date |
| --- | --- | --- |
|  |  |  |
| 792 practices  18,241,856 people |  | 2,517,068 excluded due to poor record quality or not being permanently registered. |
|  |  |  |
| 15,724,788 people |  | 5,738,766 excluded due to no data after the point that the practice met data quality thresholds for electronic recording of patient data and/or no person-time after 18^th^ birthday. |
|  |  |  |
| 9,986,022 people |  | 35,392 excluded due to having diagnosed ID prior to cohort entry and no subsequent autism diagnosis (n = 35,217), or a record of autism with no date (n = 175) |
|  |  |  |
| 22,355 people with an autism diagnosis prior to, or during follow-up |  | 9,928,260 people with no autism record at any time; 15 people with a record of autism after the end of follow-up. |
|  |  |  |
| **Autististic people with and without ID**  17,289 people contribute person-time post an autism but prior to an ID diagnosis (of whom 1,433 got a subsequent ID diagnosis prior to the end of follow-up)  6,499 people contribute person-time post an autism and an ID diagnosis. |  | **Sampling pool for identification of matched participants**  9,928,260 people with no autism record at any time.  3,965 people contribute person-time prior to an autism or an ID diagnosis. |
|  |  |  |
| 23,788 to be matched  208 had insufficient matches |  |  |
|  |  |  |
| **23,580 matched 10:1** |  | **235,800 matches** |

## eFigure 2: Simplified schematic description of steps to apply EDS to identify the matched groups & start dates

The schematics below depict a simplified version of EDS. Rows represent different individuals within a primary care practice, registered and contributing data from 2001 up until 2011 or the point at which they left the practice.

**Step 1:** We allocated person-time (after patient registration and once data quality thresholds have been met) to one of 4 categories, designated by the different colours:

|  | **2001** | **2002** | **2003** | **2004** | **2005** | **2006** | **2007** | **2008** | **2009** | **2010** |  |  | **Key** |  |  |
| --- | --- | --- | --- | --- | --- | --- | --- | --- | --- | --- | --- | --- | --- | --- | --- |
| **A** | **.** |  |  |  |  | **X** |  |  |  |  |  | **.** | **Date of registration** | | |
| **B** | **.** | **X** |  |  |  | **!** |  |  |  |  |  | **X** | **Autism diagnosis** | | |
| **C** | **.** |  |  |  |  |  |  | **X** |  |  |  | **!** | **ID diagnosis** | | |
| **D** | **.** |  |  |  |  |  |  |  |  |  |  |  | **Autism no ID** | | |
| **E** | **.** |  |  | **!** |  |  |  | **X** |  |  |  |  | **Autism with ID** | | |
| **F** | **.** |  |  |  |  |  |  |  |  |  |  |  | ***Potentially* eligible person-time** | | |
| **G** | **.** |  |  |  |  |  |  | **X!** |  |  |  |  | ***Ineligible person-time*** | | |
| **H** | **.** |  |  |  |  |  |  |  |  |  |  |  |  | | |

*B received was diagnosed with ID after being diagnosed autistic, so contributes person-time to the autism/ID- and to the autism/ID+ groups.*

**Step 2:** For each autistic person who had follow-up time without a concurrent ID record (A, B, and C), we identified a set of matched people who were in the database & had neither an autism nor an ID record on the date of the autistic person’s diagnosis. We gave them the same start (“index”) date as their autistic counterpart (the date of the autistic person’s autism diagnosis).

|  | **2001** | **2002** | **2003** | **2004** | **2005** | **2006** | **2007** | **2008** | **2009** | **2010** |  |  |  |  |  |
| --- | --- | --- | --- | --- | --- | --- | --- | --- | --- | --- | --- | --- | --- | --- | --- |
| **A** | **.** |  |  |  |  | **X** |  |  |  |  |  |  |  |  |  |
| **P** | **.** |  |  |  |  |  |  |  |  |  |  |  |  |  |  |
| **Z** | **.** |  |  |  |  |  |  |  |  |  |  |  |  |  |  |
| **B** | **.** | **X** |  |  |  | **!** |  |  |  |  |  |  |  |  |  |
| **A** | **.** |  |  |  |  | **X** |  |  |  |  | *Note that A, who is later diagnosed autistic, is sampled as a match for B, and is censored from the comparison group prior to receiving their autism diagnosis.* | | | | |
| **H** | **.** |  |  |  |  |  |  |  |  |  |  |  |  |  |  |
| **C** | **.** |  |  |  |  |  |  | **X** |  |  |  |  |  |  |  |
| **X** | **.** |  |  |  |  |  |  |  |  |  |  |  |  |  |  |
| **P** | **.** |  |  |  |  |  |  |  |  |  |  |  |  |  |  |

**Step 3:** For each autistic person who had follow-up time *with* a concurrent ID record (B, E, and G), we sampled a new set of matches by identifying people who were in the database & had neither an autism nor an ID record on the date of the autistic person’s diagnosis. We gave them the same start (“index”) date as their autistic counterpart (the latest of the date of their autism or ID diagnosis).

|  | **2001** | **2002** | **2003** | **2004** | **2005** | **2006** | **2007** | **2008** | **2009** | **2010** |  |
| --- | --- | --- | --- | --- | --- | --- | --- | --- | --- | --- | --- |
| **B** | **.** | **X** |  |  |  | **!** |  |  |  |  |  |
| **U** | **.** |  |  |  |  |  |  |  |  |  |  |
| **M** | **.** |  |  |  |  |  |  |  |  |  |  |
| **E** | **.** |  |  | **!** |  |  |  | **X** |  |  |  |
| **S** | **.** |  |  |  |  |  |  |  |  |  |  |
| **X** | **.** |  |  |  |  |  |  |  |  |  |  |
| **G** | **.** |  |  |  |  |  |  | **X!** |  |  |  |
| **L** | **.** |  |  |  |  |  |  |  |  |  |  |
| **K** | **.** |  |  |  |  |  |  |  |  |  |  |

This method allowed us to identify matched participants and start-dates for our cohort to calculate person-years at risk without introducing immortal time bias.

## eFigure 3: Identification of deaths

| 259,380 individuals |  | 1,455 deaths identified based on information from IMRD |
| --- | --- | --- |
|  |  |  |
| Additional search of medical records for codes indicating death, e.g. “cause of death”, “patient died”, “post mortem exam” |  | 55 further deaths identified that were not identified by IMRD because they had not been flagged as transferred out of the practice due to their death. |
|  |  |  |
| Medical and prescribing records for all deceased individuals checked to ensure no consulting/prescribing > 6 months after the date of death. |  | 5 individuals had records of continued consulting/prescribing after their supposed death, suggesting that their death records were erroneous. These individuals were recoded as not deceased at cohort exit. |
|  |  |  |
|  |  | 1505 deaths identified |
