# Supplementary material for: Estimating life expectancy and years of life lost for autistic people in the UK: a matched cohort study
Source: Lancet Reg Health Eur. 2023 Nov 23;36:100776. doi: 10.1016/j.lanepe.2023.100776 (PMC10769892; doi:10.1016/j.lanepe.2023.100776)
Supplement: Supplementary Methods [file mmc4.docx]

**Supplemental methods: Estimating life expectancy and years of life lost for autistic people in the UK: a matched cohort study**

***Exposure density sampling***^1^

A diagram of the sampling process is provided in eFigure 2. For autistic individuals without concurrent ID, their index date (i.e. the beginning of follow-up time) was the latest of their date of diagnosis, their date of registration at the practice + 6 months, and the date that their practice met record-keeping quality criteria. Autistic people who received an ID diagnosis during the study period were censored from the autism without ID cohort on the day prior to their ID diagnosis and joined the autism with ID group on the date of their ID diagnosis, (their new index date). They were also assigned a new set of matched participants. Those with a prior or concurrent ID diagnosis were allocated to the autism with ID group. Their index date (beginning of follow-up) was the latest of the date of their latter diagnosis (of autism or ID), their date of registration at the practice + 6 months, and the date that their practice met record-keeping quality criteria.

Age, sex, and general practice-matched comparison individuals were sampled from those registered at the practice at least six months prior to the autistic person's index date. Matched comparison individuals were assigned the same index date as their matched autistic counterparts. If a person was sampled as a comparison individual and was then diagnosed autistic or with ID during follow-up, they were censored from the comparison group the day before their diagnosis.

Unless any of the aforementioned conditions were met, follow-up ended on the earliest of each person's recorded date of death, the date that they were transferred out of the practice, or the last date that their practice contributed data to IMRD.

***Acceptable mortality recording***

We included person-time subsequent to each practice reaching the threshold for both Acceptable Computer Usage (ACU)^2^ and Acceptable Mortality Recording (AMR).^3^ The date at which the practice achieves ACU is the date at which the average annual rates of recording included least one medical record, one alternative health data record, and two prescription records per patient.^2^ The date at which the practice achieves AMR is determined based on a review of trends in death reporting for each practice against the predicted number of deaths given the practice’s demographics based on national statistics.^3^ IMRD provides a date from which the practice is reporting mortality in line with these statistics.

***Identifying deaths***

A flow chart describing identification of deaths is provided as eFigure 3. We used information about deaths provided by IMRD, which takes into account information about the person’s registration status at the practice, and codes or text entries providing information about a death. 1,455 deaths were identified based on information from IMRD. We performed an additional search for codes indicating death (Supplemental file^[[1]](#footnote-1)^), which identified 55 additional deaths (omitted by IMRD because the patient’s registration status did not indicate that they had been transferred out of the practice due to their death). For these individuals, we used the earliest of the first instance of a Read code pertaining to the death, or the person’s transfer out date, as the date of death. We counted records as deaths that occurred up to six months after the date that the person’s records were transferred out of the practice, to allow for administrative delays in recording. We further validated the recording of all deaths by checking for non-administrative medical or prescribing records more than six months after the supposed date of death. This check identified five people who had been assigned a code suggesting they had died, but had continued to receive prescriptions and had records of consultations, suggesting that they were in fact alive. Therefore, these individuals were not coded as being deceased on their date of cohort exit (the date of their supposed death). Thus, the overall number of identified deaths was 1505.

As a sensitivity analysis, we used a list of codes to identify possible deaths.^[[2]](#footnote-2)^ We identified possible deaths by finding occurrences of these codes that were up to 30 days before the date that the person was transferred out of the practice (suggesting that the potentially fatal event may have resulted in death), or up to six months after the date that the person was were transferred out.

We repeated the main analyses additionally including 48 additional possible deaths. Including possible and definite deaths, there were 108 deaths in autistic people without ID (0.63% of the sample), and 132 (2.05% of the sample in autistic people with ID). Demographics and information about co-occurring conditions in deceased autistic people with and without ID plus their respective comparison groups can be found in eTables 3 & 4. Adding these additional possible deaths had a negligible impact on mortality rates (see eTable 5) and life expectancy (see eTable 6).

**References**

1 Ohneberg K, Beyersmann J, Schumacher M. Exposure density sampling: Dynamic matching with respect to a time‐dependent exposure. *Stat Med* 2019; **38**: 4390–403.

2 Horsfall L, Walters K, Petersen I. Identifying periods of acceptable computer usage in primary care research databases. *Pharmacoepidemiol Drug Saf* 2013; **22**. DOI:10.1002/pds.3368.

3 Maguire A, Blak BT, Thompson M. The importance of defining periods of complete mortality reporting for research using automated data from primary care. *Pharmacoepidemiol Drug Saf* 2009; **18**. DOI:10.1002/pds.1688.

1. Death-related Read codes were identified by searching for codes containing the words “died”, “death”, “dead”, “decease”, “fatal”, “decedent”, “inquest”, “mortem”, “coroner”; and then excluding codes that pertained to someone else having died. [↑](#footnote-ref-1)
2. Possible death Read codes were identified by searching for codes containing the words “suicide”, “drowning”, “drowned”, “suffocat”, “hanging”, “hanged”, “murder”, “arrest”, “overdose”; and then excluding codes that were ambiguous or pertained to someone else. Codes indicating suicide and self-injury from an existing code-list were also included, with codes pertaining to specific forms of self-mutilation excluded. [↑](#footnote-ref-2)
